# Multimodal Analysis of STRADA Function in Brain Development

**DOI:** 10.3389/fncel.2020.00122

**Published:** 2020-05-08

**Authors:** Louis T. Dang, Katarzyna M. Glanowska, Philip H. Iffland II, Allan E. Barnes, Marianna Baybis, Yu Liu, Gustavo Patino, Shivanshi Vaid, Alexandra M. Streicher, Whitney E. Parker, Seonhee Kim, Uk Yeol Moon, Frederick E. Henry, Geoffrey G. Murphy, Michael Sutton, Jack M. Parent, Peter B. Crino

**Affiliations:** ^1^Department of Neurology, Michigan Medicine, Ann Arbor, MI, United States; ^2^Department of Pediatrics, Michigan Medicine, Ann Arbor, MI, United States; ^3^Michigan Neuroscience Institute, Michigan Medicine, Ann Arbor, MI, United States; ^4^Department of Neurology, University of Maryland School of Medicine, Baltimore, MD, United States; ^5^Department of Neurosurgery, Weill-Cornell Medical Center, New York, NY, United States; ^6^Louis Katz School of Medicine, Temple University, Philadelphia, PA, United States; ^7^Department of Molecular, and Integrative Physiology, Michigan Medicine, Ann Arbor, MI, United States; ^8^Neurology Service, VA Ann Arbor Healthcare System, Ann Arbor, MI, United States

**Keywords:** mTOR, megalencephaly, epilepsy, iPSC, mouse, seizure

## Abstract

mTORopathies are a heterogeneous group of neurological disorders characterized by malformations of cortical development (MCD), enhanced cellular mechanistic target of rapamycin (mTOR) signaling, and epilepsy that results from mutations in mTOR pathway regulatory genes. Homozygous mutations (del exon 9–13) in the pseudokinase STE20-related kinase adaptor alpha (*STRAD-α*; *STRADA*), an mTOR modulator, are associated with Pretzel Syndrome (PS), a neurodevelopmental disorder within the Old Order Mennonite Community characterized by megalencephaly, intellectual disability, and intractable epilepsy. To study the cellular mechanisms of STRADA loss, we generated CRISPR-edited *Strada* mouse N2a cells, a germline mouse *Strada* knockout (KO−/−) strain, and induced pluripotent stem cell (iPSC)-derived neurons from PS individuals harboring the *STRADA* founder mutation. *Strada* KO *in vitro* leads to enhanced mTOR signaling and iPSC-derived neurons from PS individuals exhibit enhanced cell size and mTOR signaling activation, as well as subtle alterations in electrical firing properties e.g., increased input resistance, a more depolarized resting membrane potential, and decreased threshold for action potential (AP) generation. *Strada*−/− mice exhibit high rates of perinatal mortality and out of more than 100 litters yielding both WT and heterozygous pups, only eight *Strada*−/− animals survived past P5. *Strada*−/− mice are hypotonic and tremulous. Histopathological examination (*n* = 5 mice) revealed normal gross brain organization and lamination but all had ventriculomegaly. Ectopic neurons were seen in all five *Strada*−/− brains within the subcortical white matter mirroring what is observed in human PS brain tissue. These distinct experimental platforms demonstrate that STRADA modulates mTOR signaling and is a key regulator of cell size, neuronal excitability, and cortical lamination.

## Introduction

“Pretzel syndrome” (PS) also known as polyhydramnios, megalencephaly, symptomatic epilepsy syndrome (PMSE; OMIM#611087) is an autosomal recessive neurodevelopmental disorder characterized by megalencephaly (ME), severe developmental delay, and medically intractable epilepsy in association with intrauterine polyhydramnios and renal dysfunction (Puffenberger et al., 2007). Post-mortem histopathological examination of a single brain specimen revealed enlarged brain size, enhanced cortical neuronal size, and heterotopic neurons in the subcortical white matter, with preserved gyral patterning (Puffenberger et al., 2007). In all Mennonite individuals, PS is caused by a homozygous founder deletion spanning exons 9–13 of the STE20-related kinase adaptor alpha (*STRADA*) gene (17q23.3) although additional non-Mennonite (non-founder) *STRADA* variants associated with PS have been reported e.g., a consanguineous Asian pedigree, c.842dupA, p.D281fs (Bi et al., 2016), and a consanguineous Turkish pedigree, c.891dupC; p.C298Lfs* (Evers et al., 2017), demonstrating that *STRADA* is relevant outside of the Mennonite community as an epilepsy and ME gene.

STRADA modulates the mechanistic target of rapamycin (mTOR) pathway as part of the LKB1/STRADA/MO25 complex that signals *via* AMPK (Hawley et al., 2003), to TSC1/TSC2/TBC1D7 and then to mTOR in response to many upstream cellular cues including cellular ATP levels (Crino, 2016). STRADA is a pseudokinase that augments LKB1 kinase activity when bound to LKB1 (Boudeau et al., 2003). In the absence of STRADA, LKB1 has minimal kinase activity, and thus it cannot phosphorylate one of its primary substrates, AMPK. Interestingly, knockout of *Lkb1* in mice leads to abnormal brain development (Asada et al., 2007; Barnes et al., 2007) yet variants in *LKB1* and *MO25* are not linked to human epilepsy or cortical malformations.

shRNA-mediated knockdown (KD) of *Strada* in mouse neural progenitor cells *in vitro* causes rapid activation of the mTOR signaling cascade and enhanced cell size, a phenotype commonly seen with activated mTOR pathway signaling (Orlova et al., 2010). *Strada* KD causes altered cell polarity, disorganized Golgi assembly, and altered motility, effects that can be prevented with the mTOR complex 1 (mTORC1) inhibitor rapamycin (Parker et al., 2013). *Strada* KD in fetal mouse brain by *in utero* electroporation at embryonic day 14–15 causes a cortical lamination defect with heterotopic neurons in the white matter, an effect that can be prevented with the mTOR inhibitor rapamycin. Interestingly, germline* Strada* knockout (KO) is a perinatal lethal phenotype with death on or around post-natal day 2 (Veleva-Rotse et al., 2014). These animals exhibit defects in axonogenesis but brain structure is otherwise intact. Finally, the treatment of PS individuals with rapamycin (sirolimus) can alter seizure frequency but does not affect intellectual disability (Parker et al., 2013).

To more fully define the role of STRADA in brain development, we have generated a *Strada* KO mouse strain carrying the same mutation (del exon 9–13) as humans with PS and we have generated induced pluripotent stem cells (iPSCs) from fibroblasts obtained from PS patients to derive neurons for morphological and electrophysiological analysis.

## Materials and Methods

### CRISPR/Cas9 Construct Generation and Validation

Guide RNA targeting the spCas9 endonuclease to regions in the mouse genome encoding *Strada* (-AGTCGCCATTGGAAGGCCGGAGG-) were calculated *in silico* using ChopChop software (chopchop.cbu.uib.no). A scramble gRNA (-GACTACCAGAGCTAACTCA-) was used as a transfection and gRNA control. *In silico* guide RNAs were then assembled into oligonucleotides (Integrated DNA Technologies, Coralville, IA, USA), annealed using ligase buffer (Promega, Madison, WI, USA) at 98°C for 5 min. Annealed gRNA was then sub-cloned into PX330-based plasmid (addgene #48138) using Golden Gate Assembly containing a mCherry reporter linked to Cas9 *via* a T2a multicistronic element. Plasmid assembly was confirmed by Sanger sequencing (Genewiz, South Plainfield, NJ, USA).

To validate that our gRNA containing CRISPR/Cas9 plasmid created indels in our regions of interest, DNA from *Strada*, and scramble FAC-sorted cells lines (as described below) as well as wildtype (WT) N2aC was assayed for mismatched DNA pairs (EnGen Mutation Detection Kit; New England Biolabs, Ipswich, MA, USA) with PCR primers targeted towards our genomic region of interest (Integrated DNA Technologies, Coralville, IA, USA).

### Cell Culture and Establishment of CRISPR KO Cell Lines

Neuro2a cells (N2aC; Sigma–Aldrich, St. Louis, MO, USA) were cultured in complete medium consisting of EMEM (Invitrogen, Carlsbad, CA, USA) supplemented with 10% FBS (Invitrogen, Carlsbad, CA, USA). To create stable CRISPR/Cas9 edited cell lines, N2aC were transfected using Lipofectamine LTX with Plus reagent (Thermo Fisher Scientific, Waltham, MA, USA) and 30 μg of plasmid diluted in 300 μl Opti-MEM (Invitrogen, Carlsbad, CA, USA) for 48 h. Co-transfection experiments were performed by using 30 μg of each plasmid. After 48 h of transfection, cells were trypsinized (0.25%), centrifuged, washed with ice-cold PBS, passed through a cell strainer into a 5 ml conical tube and assayed by flow cytometry (University of Maryland School of Medicine Flow Cytometry Core) for sorting based on mCherry (Cas9) fluorescence (BD FACSAria II cell sorter; Becton Dickinson and Company, Franklin Lakes, NJ, USA). mCherry+ sorted cells were placed into PBS containing 1% serum until re-plating. Cells were re-plated in complete media and grown to confluence.

### Immunocytochemistry

N2aC were fixed in 4% PFA at room temperature (RT) for 20 min and then permeabilized in phosphate-buffered saline (PBS) containing 0.3% Triton X-100 (Thermo Fisher Scientific, Waltham, MA, USA). Cells were blocked for 2 h at RT in 5% normal goat serum (Jackson ImmunoResearch, West Grove, PA, USA). Cells were incubated in one of the following primary antibodies in blocking solution containing 5% normal serum at 4°C overnight: rabbit monoclonal to phospho-S6 ribosomal protein (Ser235/236, 1:1,000; Cell Signaling).

### Human Fibroblasts Isolation and Culture

PS patient (*n* = 2) and control (*n* = 2) human fibroblasts were obtained from skin-punch biopsies at the Clinic for Special Children (CSC) in Lancaster, PA, USA, following informed consent. Skin biopsies were performed following approved Institutional Review Board protocols at the University of Pennsylvania and Temple University (where the study was initiated), and Lancaster General Hospital (Lancaster, PA, USA). Parents provided informed consent before their child’s participation.

Fibroblasts were extracted from tissue samples by incubation in 0.25% Trypsin/EDTA (Gibco) overnight at 4°C. The next day, the epidermis was removed, and dermis was digested with Collagenase P (Roche) buffered in 130 mM sodium chloride (Sigma–Aldrich), 10 mM calcium acetate (Sigma–Aldrich), and 20 mM HEPES buffer for 30 min at 37°C. Then 0.5% Trypsin/EDTA (Gibco) was added, and the mixture was incubated at 37°C for an additional 10 min before neutralization with fibroblast culturing media, composed of DMEM supplemented with 10% FBS (Sigma–Aldrich), 10 mM HEPES buffer, 1% penicillin/streptomycin (10,000 U/ml penicillin, 10 mg/ml streptomycin stock), and 1% fungizone. Fibroblasts were pelleted through centrifugation for 5 min at 1,500 rpm, and the pellet was resuspended in fibroblast culturing media to obtain the desired cells.

### Fibroblast Reprogramming and iPSC Culture

Fibroblasts were cultured in DMEM, 10% FBS, 1X L-glutamine, 1 mM MEM non-essential amino acids (NEAA), 50 U/ml penicillin, and 50 μg/ml streptomycin (all from Life Technologies, Carlsbad, CA, USA) at 37°C and 5% CO_2_. For retroviral reprogramming (Liu et al., 2013), viral stocks were obtained using GP2–293 packaging cells (Clontech, Mountain View, CA, USA) and retroviral vectors encoding Oct3/4, Sox2, Klf4 and c-Myc on a pMXs backbone (Addgene, Cambridge, MA). Fibroblasts plated in 6-well plates (30,000 per well) were transduced with retroviruses, followed by a second round of transduction the next day. After 4 days, fibroblasts were passaged onto mouse embryonic fibroblasts (MEFs, GlobalStem, Rockville, MD, USA) using 0.25% trypsin (Life Technologies) and switched 1 day later to a stem cell medium containing: DMEM/F12, 20% knock-out serum replacement, 1X L-glutamine, 1 mM MEM non-essential amino acids, 50 U/ml penicillin, 50 μg/ml streptomycin, 4 μg/ml FGF2 (Life Technologies) and 100 μM β-mercaptoethanol (Sigma–Aldrich, St., Louis, MO, USA). Fibroblasts from PS subject 2 were reprogrammed using an episomal method (Okita et al., 2011). Fibroblasts were electroporated with plasmids pCXLE-hOCT3/4-shp53-F, pCXLE-hSK, and pCXLE-hUL (Addgene# 27077, 27078, and 27080, respectively; gift from Shinya Yamanaka). Fibroblasts were grown in TeSR-E7 (StemCell Technologies, Vancouver, BC, USA) media for 17–21 days before picking single iPSC colonies.

iPSC colonies appeared and were manually picked and passaged onto MEFs and grown with stem cell medium or plated onto Matrigel (1:250 dilution in DMEM/F12; BD Biosciences, San Jose, CA, USA) and grown with mTeSR1 (StemCell Technologies). iPSCs were passaged weekly using either 5 mM AccuGENE EDTA (diluted 1:100 in DPBS without calcium or magnesium; Lonza, Basel, Switzerland), Versene (Life Technologies), or Dispase (Thermo Fisher Scientific, Waltham, MA, USA). For passaging with EDTA or Versene, culture medium was supplemented with 10 μM Y-27632 ROCK inhibitor (EMD Millipore, Darmstadt, Germany) for 24 h (Narumiya et al., 2000). After six passages the cells were evaluated for pluripotency by immunocytochemistry (ICC) and embryoid body differentiation. For embryoid body experiments, iPSC colonies were grown in suspension for a week, passaged onto 0.1% porcine type A gelatin (Sigma) for another week and processed for ICC. iPSC samples were karyotyped by Cell Line Genetics (Madison, WI, USA).

### Neuronal Differentiation

iPSCs were dissociated with Versene or Accutase (Innovative Cell) and plated on Matrigel or CELLstart (Life Technologies). Upon reaching 95% confluence, the culture medium was switched to 3N medium (Shi et al., 2012), a 1:1 mix of N2 (DMEM/F12, 1X N2, 5 μg/ml insulin, 1 mM L-glutamine, 1X MEM NEAA, 100 μM 2-mercaptoethanol, 50 U/ml penicillin, 50 μg/ml streptomycin) and Neurobasal (with 1X B27, 1 mM L-glutamine, 50 U/ml penicillin and 50 μg/ml streptomycin) supplemented with 10 μM SB431542 and 1 μM Dorsomorphin (both from Tocris, Bristol, UK). The medium was changed daily for 12 days, then the cell monolayer was broken into aggregates of 300–500 cells with Dispase. The aggregates were resuspended in 3N media and then plated on Matrigel and grown in 3N media without supplements. Culture media was exchanged every 48 h. After neural rosettes appeared, the medium was supplemented with 20 ng/ml of FGF2 for 4 days and the aggregates were passaged with Dispase and cultured in unsupplemented 3N media. When neurons appeared at the edges of the colonies, cells were dissociated with Accutase and replated on Matrigel for immunocytochemistry.

### Immunocytochemistry and Morphometric Analysis of iPSC Derived Neurons

Cells were plated on Matrigel and cultured for 1 week (for iPSCs or embryoid bodies) or 3–5 weeks (for neuronal cultures). The cells were rinsed with PBS and fixed in 4% paraformaldehyde (PFA) for 30 min, then washed twice in PBS at RT for 10 min. Cells were permeabilized in 0.2% Triton X-100 (Sigma; diluted in PBS) for 5 min at RT, and then blocked in a buffer containing 10% normal goat serum, 0.05% Triton X-100 and 1% bovine serum albumin (Sigma) diluted in PBS for 1 h at RT. Primary antibodies (Table 1) were resuspended in blocking buffer and incubated with the cells overnight at 4°C. After three washes (10 min each) with PBS, cells were incubated in secondary antibodies (Table 1) at a 1:400 dilution in blocking buffer for 90 min at RT. Nuclei were stained with bisbenzamide (Invitrogen; 1:5,000 dilution in PBS) for 5 min at RT. Cells were then washed three times with PBS. Images were obtained with a Leica DMI 6000B epifluorescent microscope using the Leica Application Suite Advanced Fluorescence software (Leica Microsystems Inc., Buffalo Grove, IL, USA) and analyzed using ImageJ (NIH, Bethesda, MD, USA). For quantification of P-S6 immunoreactivity, ROIs were created around the somatic region of control and PS cells using the MAP2 channel, and average non-zero pixel intensity in the P-S6 channel was determined for each cell. Somatic P-S6 intensities for each cell were normalized to the average control value and expressed as a percent of control. Changes in P-S6 expression were analyzed using an unpaired student’s *t*-test (two-tailed).

**Table 1:** Primary and secondary antibodies used for ICC.

Target	Host	Company	Catalog Number	Dilution
**Primary antibodies**
NANOG	Rabbit	Abcam	ab21624	1:500
OCT3/4	Goat	Santa Cruz Biotechnology	sc-8628	1:100
SSEA4	Mouse	DSHB	MC-813–70	1:200
α Feto-protein	Rabbit	Dako/Agilent	A0008	1:500
Smooth muscle actin	Mouse	Abcam	ab5694	1:1,000
SOX2	Rabbit	Millipore	AB5603	1:1,000
p-S6 (S235/236)	Rabbit	Cell Signaling	4858	1:250
βIII-tubulin (TuJ1)	Mouse	Covance	MMS-435P	1:2,000
Cux1	Rabbit	Santa Cruz Biotechnology	Sc-13024	1:50
βIII-tubulin (TuJ1)	Mouse	Biolegend	801202	1:2,000
Doublecortin	Rabbit	Abcam	ab18723	1:500
Map2abc	Rabbit	Cell Signaling	4542S	1:500
Map2ab	Mouse	Sigma	M2320	1:500
GABA	Rabbit	Sigma	A2052	1:500
vGlut1	Guinea pig	Millipore	AB5905	1:200
GFAP	Rabbit	Dako/Agilent	Z0334	1:500
**Secondary antibodies**				
488 anti-rabbit IgG	Goat	Invitrogen	A-11034	1:400
488 anti-mouse IgG	Goat	Invitrogen	A-11001	1:400
488 anti-goat IgG	Donkey	Invitrogen	A-11055	1:400
594 anti-rabbit IgG	Goat	Invitrogen	A-11037	1:400
594 anti-mouse IgG	Goat	Invitrogen	A-11032	1:400
FITC anti-guinea pig IgG	Goat	Millipore	AP108F	1:200

To analyze neuronal differentiation, neuronal cultures were stained for doublecortin (DCX) and neuron-specific-βIII-tubulin (TUBB3) to detect immature neurons, and microtubule-associated protein 2ab (MAP2ab) to detect mature neurons (Table 1). Samples stained for mature and immature neuronal markers were used for soma size measurement by tracing the soma of individual neurons in ImageJ. A calculated area was then obtained based on the scale bar value of the images.

### Data Quantification and Statistical Analysis

Cell lysates were electrophoresed and probed with the following primary antibodies: rabbit monoclonal to phospho-S6 ribosomal protein (P-S6; Ser235/236, 1:1,000; Cell Signaling), rabbit polyclonal anti-LYK5 (recognizes STRADA; 1:500; Abcam), and rabbit monoclonal to GAPDH (1:1,000; Cell Signaling). At least two separate samples were taken for each cell line per differentiation and processed as described in the “Materials and Methods” section. WB films were digitized and the images analyzed using the Gel Analysis function of ImageJ. Bands for P-S6 were measured and the values normalized to the signal of GAPDH (used as a loading control) for each sample using a fixed window size. A second normalization was performed to the sample with the highest ratio in the same WB film. Average ratios per iPSC cell line were compared using ANOVA and planned contrasts, with statistical significance set at 0.05, in R Core Team (2014). For soma size measurements, 2 separate differentiations were performed per experimental group, and neuronal cultures were stained for DCX, TUBB3, and MAP2 antibodies as described above. For each differentiation and experimental group 5–10 random fields-of-view (FOV) were imaged, and 100 cells positive for each marker selected for measurement. The soma of each selected cell was traced using ImageJ and the resulting areas were pooled for each experimental group. Statistical comparisons were performed using unpaired, two-tailed Student’s *t*-test. Patch-clamp recording data were analyzed for significance using unpaired, two-tailed Student’s *t*-test to compare passive and active electrophysiological properties between experimental groups. The proportions of spontaneously active neurons between control and PS groups were compared using the two-tailed Chi-square test.

### Whole-Cell Patch-Clamp Recordings

Neuronal cultures were plated on glass-bottom dishes (MatTek, Ashland, MA, USA) or coverslips coated with Matrigel and cultured in BrainPhys medium (Bardy et al., 2015) for 8–12 weeks before recordings were made. Immediately before electrophysiological recordings, dishes/coverslips with either patient or control neurons were transferred to a recording chamber filled with fresh, CO_2_-saturated BrainPhys and stabilized for at least 10 min. In our experience, neurons remain healthy in Hibernate A for approximately 2–3 h outside of the incubator, and all electrophysiological recordings from a given culture were completed within 1.5 h.

Recording micropipettes were pulled from capillary glass (type 7052, outer diameter/inner diameter 1.65/1.1 mm; World Precision Instruments, Sarasota, FL, USA) using a Flaming/Brown P-97 pipette puller (Sutter Instruments, Novato, CA, USA) to obtain pipettes with a resistance of 3.0–5.0 MΩ. Whole-cell recordings were made in voltage-clamp and current-clamp mode of an Axon amplifier with pClamp 10.0 software (Molecular Devices LLC, Sunnyvale, CA, USA) and digitized using a Digidata 1440A digitizer (Molecular Devices). Data were filtered at 3 kHz and digitized at 20 kHz. Neurons were visualized on an upright microscope (Olympus, Center Valley, PA, USA) under differential interference contrast using an Olympus OLY-150 IR CCD camera. Micropipettes were filled with an internal solution containing (in mM): 120 potassium gluconate, 20 KCl, 10 HEPES, 0.2 EGTA, 2 MgCl*6H_2_O, 4 Na2 ATP, 0.3 Tris-GTP, 7 phosphocreatine, pH adjusted to 7.25 with KOH. Passive properties (input resistance, series resistance, and capacitance) were monitored in voltage-clamp mode throughout recordings and only cells with stable properties were further analyzed.

Current-clamp recordings were performed to study intrinsic excitability and action potential (AP) firing properties. For some recordings, neuronal cultures were previously transfected with a lentiviral vector carrying a GFP reporter driven by the CaMKIIα promoter to aid in the prospective identification of mature neurons. After establishing a whole-cell configuration and measuring passive properties under voltage clamp, the recording mode was switched to current-clamp and resting membrane potential was recorded. Some neurons exhibited spontaneous AP firing while others displayed a depolarized membrane potential preventing them from generating APs, likely due to the inactivation of sodium channels. Therefore, a continuous injection of hyperpolarizing current was applied until they stopped firing and their membrane voltage reached approximately −60 to −65 mV to study their intrinsic excitability. To ensure proper comparisons between neurons resting at variable potentials and having different levels of spontaneous activity, all intrinsic excitability studies were performed on cells hyperpolarized to the same level *via* hyperpolarizing current. Neurons were initially depolarized with a brief high amplitude current injection to evoke a single AP followed by a series of 1-second-long depolarizing steps of increasing amplitude to study repetitive AP firing.

Voltage clamp recordings were performed to assess the presence of sodium and potassium conductances as well as spontaneous synaptic activity. Briefly, to record Na^+^ and K^+^ currents neurons were held at −60 mV and received a series of 200 ms long voltage steps from −100 to +90 mV. Spontaneous excitatory postsynaptic currents (PSCs) were recorded in a gap-free mode for 3 min at holding potential set at −60 mV.

### Germline Mouse KO

An exon 9–13 deletion (6200 bp) homologous to the human PS locus (7304 bp deletion spanning exons 9–13) was engineered into C57/Bl6N mice with a *Neo* cassette and two *LoxP* sites (Supplementary Figure S1). Heterozygous (+/−) and homozygous (−/−) animals were bred in combination, but perinatal lethality was noted in litters following both breeding approaches; we did not generate enough viable *Strada*−/− pups for *Strada*−/− matings. Germline Strada KO (*Strada*) was confirmed by Southern blot (Supplementary Figure S2). Litters were monitored for behavioral abnormalities (movement, suckling, seizures) from P0-P30 by direct observation and continuous 12-h video-monitoring. Body masses (grams) were obtained for all live pups at select post-natal dates. Wildtype, *Strada*+/− and *Strada*−/− animals were sacrificed *via* ice anesthesia and intracardial perfusion (P0–P20) or by CO_2_ asphyxiation followed by intracardial perfusion (P21+) with PBS. Brain specimens were fixed in 4% PFA, paraffin-embedded, and sectioned at 10 microns. The sections were probed with antibodies targeting P-S6. Slides were counterstained with DAPI and imaged on a fluorescent microscope. All animal experiments were approved by the Institutional Animal Care and Use Committee of the University of Pennsylvania School of Medicine, Temple University, Philadelphia, PA, and the University of Maryland School of Medicine, Baltimore, MD, USA (where the studies were performed).

## Results

### Enhanced mTOR Signaling

**Figure 1:**
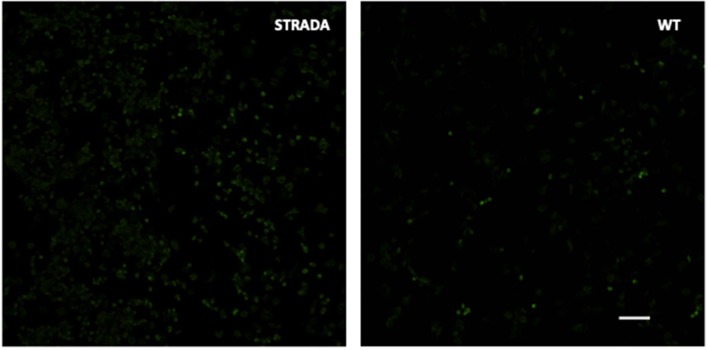
Immunocytochemistry showing enhanced phosphorylation of ribosomal S6 protein in CRISPR-edited *Strada* N2a cells vs. wildtype (WT) cells. Scale bar, 250 microns.

CRISPR KO of *Strada* in mouse N2a cells resulted in enhanced S6 phosphorylation compared with wildtype N2a cells (Figure 1) as previously demonstrated following the shRNA knockdown of *Strada* in mouse neural progenitor cells *in vitro* and *in vivo* (Orlova et al., 2010; Parker et al., 2013). As in these previous studies, there was no change in non-phosphorylated S6 levels (data not shown) following *Strada* CRISPR editing.

### iPSCs and Derived Neurons

**Figure 2:**
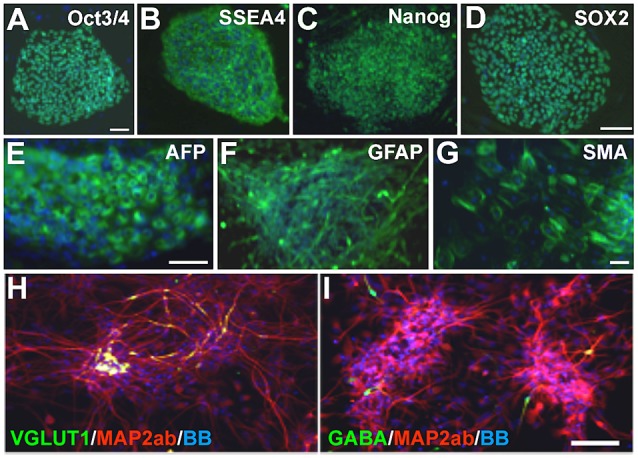
Pretzel Syndrome (PS) induced pluripotent stem cell (iPSC) pluripotency and neuronal differentiation. **(A–D)** iPSCs obtained from reprogramming PS fibroblasts were immunostained for the pluripotency markers (in green) OCT3/4 **(A)**, SSEA4 **(B)**, NANOG **(C)** and SOX2 **(D)**. **(E–G)** Embryoid bodies differentiated from PS iPSCs contained cells from all three germinal layers (green): AFP (**E**; endoderm), GFAP (**F**; ectoderm) and SMA (**G**; mesoderm). **(H,I)** Neuronal differentiation of PS iPSCs yields mostly microtubule-associated protein 2ab (MAP2ab)/vesicular glutamate transporter type 1 (VGLUT1) double-labeled **(H)** or MAP2ab-positive and gamma-aminobutyric acid-negative **(I)** mature excitatory and rare GABA+ inhibitory cortical-like neurons. Bisbenzimide nuclear stain is in blue in all panels. Scale bars: 100 μm in **(A)** for **(A–C)** and **(G)**; 75 μm in **(D)**, in **(E)** for **(E,F)**, and in **(I)** for **(H,I)**.

Patient 1: fibroblasts were obtained from a 4-year-old child with PS who was non-verbal and suffered from recurrent seizures. Germline *STRADA* mutation was confirmed by Sanger sequencing. Patient 2: fibroblasts were obtained from a 3-year-old child with genotype-confirmed PS, with intractable seizures and severe intellectual disability. Fibroblasts from two separate healthy 1-year-old individuals as well as commercially available foreskin fibroblasts (GlobalStem) were used to generate control iPSCs. Fibroblasts were reprogrammed to iPSCs by retroviral transduction or episomally with plasmid electroporation of OCT3/4, KLF4, SOX2, and c-MYC (see “Materials and Methods” section). Two iPSC clones were expanded from each of the patients’ fibroblasts and 2–3 from each control. All clones showed immunoreactivity for the pluripotency markers OCT3/4, SSEA4, NANOG and SOX2 (Figures 2A–D, and data not shown). The ability of the clones to differentiate into all three germ layer derivatives was tested with an embryoid body assay. All iPSCs were differentiated into cells expressing either α-fetoprotein (endoderm), smooth muscle actin (mesoderm) and βIII-tubulin or glial fibrillary acidic protein (GFAP; ectoderm; Figures 2E–G and data not shown), confirming pluripotency. The karyotype of all cell lines was normal (data not shown).

### Neuronal Differentiation

iPSCs were differentiated into a neuroepithelial monolayer using dual SMAD inhibitors. Neuroepithelial cells were mechanically disrupted and passaged in a culture medium containing Neurobasal, DMEM/F12, N2, B27, and insulin to generate neural rosettes, and then differentiated into neurons. The resulting neuronal cultures contained a mixture of neural progenitors, mature and immature neurons, and astrocytes (data not shown). After 3–5 weeks of differentiation, patient and control cultures contained both glutamatergic and GABAergic neurons, with a predominance of the former (Figures 2H,I) as expected with the dual SMAD inhibitor differentiation protocol we used.

### *STRADA* Deletion and Loss of *STRADA* Expression

**Figure 3:**
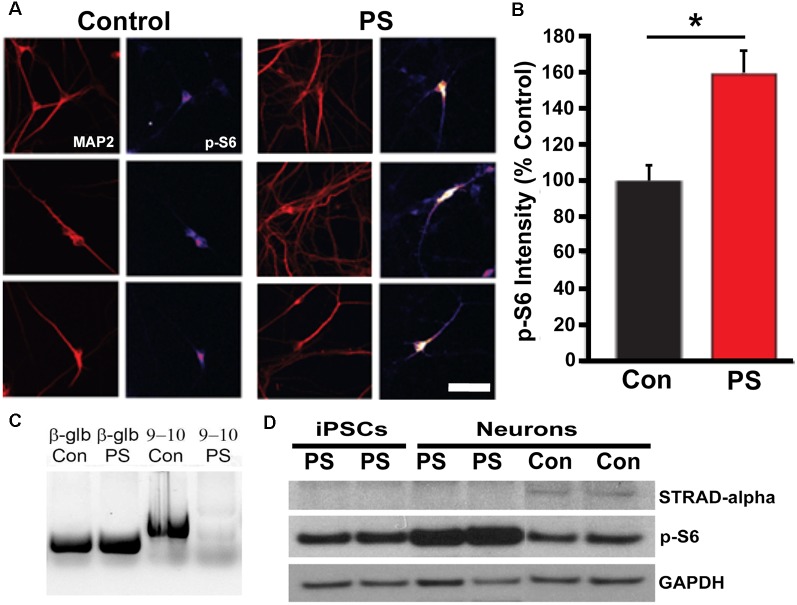
STRADA and phosphorylated ribosomal-S6 (p-S6) expression in iPSC-derived control and PS neurons. **(A)** Immunocytochemistry for p-S6 of control vs. PS iPSC-derived neurons immunolabeled for p-S6 and MAP2abc. P-S6 signal is displayed as an intensity map (fire, NIH ImageJ), Scale bar = 40 um. **(B)** Mean p-S6 immunocytochemical signal intensity of control- vs. PS-derived MAP2+ neurons. The bar graph represents mean ± SD, with values normalized to the mean of the control samples. *p < 0.05 (*t*-test). **(C)** Targeted PCR using primers spanning *STRADA* exons 9–10. Lane 1, beta-globin (β-glb) detected in control (Con) individual fibroblast DNA (250 bp product); Lane 2, β-glb detected in PS fibroblast DNA; Lane 3, detection of STRADA exons 9–10 in control fibroblast genomic DNA (598 bp product); Lane 4, absence of STRADA exons 9–10 in PMSE fibroblast genomic DNA. **(D)** Western blot for: (1) STRADA protein (top row) showing absent expression in iPSCs and neurons derived from PS fibroblasts compared with control derived neurons (Con); (2) p-S6 protein (middle row) displaying increased p-S6 in PS neurons compared to PS iPSCs and control neurons; and (3) GAPDH loading control (bottom row).

We used PCR and Western blot to confirm the expected deletion in the *STRADA* gene and loss of protein expression in cells from PS individuals targeted PCR using primers spanning *STRADA* exons 9–11 failed to generate an amplicon from the PS fibroblasts thus confirming the deletion of exons 9–13 that is common to all Mennonite PS individuals, whereas STRADA amplicons were generated from control fibroblasts (Figure 3C). Amplicons for each primer set were also generated from control lymphoblasts and post-mortem control brain tissue (data not shown). Western assay did not detect STRADA protein in PS iPSCs or neurons but did detect STRADA protein in control neurons (Figure 3D). These data confirm the exon 9–13 deletion in the PS patient fibroblasts that were used to derive our iPSCs, and the loss of STRADA protein expression in iPSCs and derived neurons.

### mTOR Activation and Cytomegaly

To assay for increased mTOR activation in PS iPSCs and neurons, we measured ribosomal S6 protein phosphorylation (S235/236). We found increased phosphorylation of S6 in PS-derived neurons compared with control cells by both Western blot (*p* = 0.02) and ICC (*p* < 0.05; Figures 3A,B,D). There was no change in non-phosphorylated S6 levels (data not shown). These results indicate that the effects of STRADA deletion on mTOR signaling in our derived neurons are similar to those seen in animal models of PS, as well as human PS brain specimens and fibroblast cultures.

**Figure 4:**
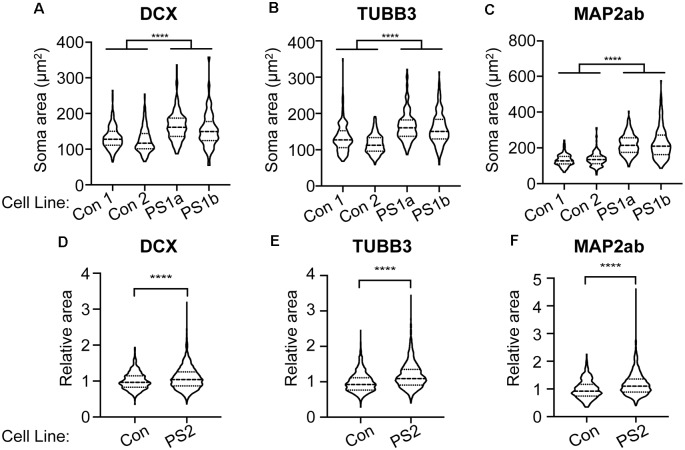
PS iPSC-derived neurons have larger soma compared to control neurons. Soma size was measured for each experimental group using markers for immature (DCX, TUBB3) and mature (MAP2ab) neurons. Violin plots with quartiles are displayed. **(A–C)** Two control cell lines (Con) and two cell lines from PS subject 1 were quantified from two independent neuronal differentiations, and the data for each genotype were combined. **(A)** Neurons labeled for DCX. Con *n* = 432 PS 1: *n* = 438. **(B)** Neurons labeled for TUBB3. Con: *n* = 426, PS 1: *n* = 431. **(C)** Neurons labeled for MAP2ab. Con: *n* = 433, PS 1: *n* = 434. **(D–F)** Neuronal differentiations were repeated using a separate control cell line (Con) and one cell line from PS subject 2 (PS 2). For each differentiation, soma size was normalized to the control neurons to account for batch to batch variation. Two independent neuronal differentiations were quantitated for DCX **(D)** and four independent differentiations were quantitated for TUBB3 **(E)** and MAP2ab **(F)**. **(D)** Neurons labeled for DCX. Con: *n* = 563, PS 2: *n* = 1,711. **(E)** Neurons labeled for TUBB3. Con: *n* = 866, PS 2: *n* = 2,430. **(F)** Neurons labeled for MAP2ab. Con: *n* = 430, PS 2: *n* = 1,944. Statistical analysis was performed using an unpaired Student’s *t*-test with Welch’s correction. ****p < 0.0001.

mTOR hyperactivity is associated with cytomegaly and cell size is enhanced in human PS brains (Puffenberger et al., 2007). To examine whether PS patient-derived cells exhibit cytomegaly, we measured soma sizes of PS and control iPSC-derived immature and mature neurons. Immature neurons were immunolabeled with either DCX or neuron-specific-βIII-tubulin (TUBB3; TuJ1 clone). We found significantly increased soma area in neurons derived from two PS patient 1 clones (PS 1a and 1b) compared to two different human control iPSC-derived neurons (Figures 4A,B). Mature PS iPSC-derived neurons immunolabeled for MAP2ab also exhibited significantly greater soma area compared to controls (Figure 4C); the differences appeared greatest in the mature neurons (note different scale bars between A/B and C in Figure 4). We also reproduced these results using iPSCs from PS subject #2 and an additional control iPSC line. Two neuronal differentiations were labeled with DCX, and four differentiations with TUBB3 and MAP2ab. With each differentiation, the sizes were normalized to the mean control soma size to control for batch variation. We again found increased soma size in the PS cells (Figures 4D–F). Taken together with the data above, these findings demonstrate that loss of *STRADA* in PS patient-derived neurons leads to cytomegaly as seen following *Strada* KD (Orlova et al., 2010) or *Strada* KO *via* CRISPR (Figure 1).

### PS Neurons Show a Subtle Electrophysiological Phenotype

**Figure 5:**
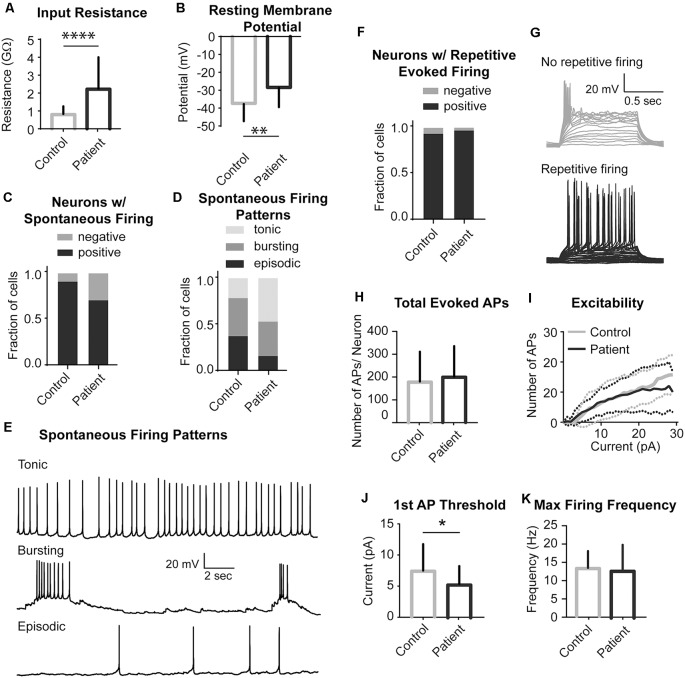
Electrophysiological properties of PS patients and control neurons. **(A)** Summary data of Input Resistance, data represented as Mean ± SEM; sample sizes: Control (gray bar) *n* = 31, Patient (black bar) *n* = 27; Unpaired Student’s *t*-test, ****p < 0.0001. **(B)** Summary data of Resting Membrane Potential, data represented as Mean ± SEM; sample sizes: Control (gray bar) *n* = 31, Patient (black bar) *n* = 27; Unpaired Student’s *t*-test, **p = 0.0016. **(C)** The fraction of neurons firing spontaneously at −40 mV, data represented as a fraction of the total cell number; black bar- neurons with spontaneous firing, gray bars- neurons with no spontaneous firing; sample sizes: Control *n* = 31, Patient *n* = 27; Fisher’s exact test, *p* = 0.0913 (n.s.). **(D)** Distribution of the pattern of spontaneous firing represented as fraction of total of all cells with spontaneous firing; sample sizes: Control = 28, Patient = 19; Chi-square test χ = 3.987, df = 2, *p* = 0.1362 (n.s.). **(E)** Example recordings of three different spontaneous firing patterns: Tonic, Bursting and Episodic. **(F)** A fraction of neurons firing repetitive evoked action potentials (APs), data represented as a fraction of the total cell number; black bar- neurons with repetitive firing, gray bar- neurons with no repetitive firing; sample sizes: control *n* = 28, Patient *n* = 26; Fisher’s exact test, *p* > 0.999 (n.s.). **(G)** Example recordings of a series of depolarizing current injections to a neuron demonstrating no repetitive firing (top gray traces) and a neuron firing repetitively (bottom black traces). **(H)** Summary data of the Total Evoked APs, data represented as Mean ± SEM; sample sizes: Control (gray bar) *n* = 27, Patient (black bar) *n* = 22; Unpaired Student’s *t*-test, *p* = 0.5634 (n.s). **(I)** Input-Output curves characterizing excitability of neurons derived from control and mutant neurons, data represented as the mean number of APs (Y-axis) evoked with a given current injection (X-axis); sample sizes: Control (solid gray line) *n* = 27, Patient (solid black line) *n* = 22, dotted lines represent the range of SEM for each experimental group; multiple *t*-tests for each current step, adjusted *p* values n.s. **(J)** Firing threshold represented as minimum current required to evoke action potential (AP) firing, data represented as Mean ± SEM; sample sizes: Control (gray bar) *n* = 26, Patient (black bar) *n* = 22; Unpaired Student’s *t*-test, *p = 0.0468. **(K)** Maximum Evoked Firing Frequency, data represented as Mean ± SEM; sample sizes: Control (gray bar) *n* = 26, Patient (black bar) *n* = 22; Unpaired Student’s *t*-test, *p* = 0.8784 (n.s.). For all recordings, no differences were found between the two patient lines and they were pooled.

We next investigated whether *STRADA* deletion alters neuronal excitability in a manner that might explain the severe seizure phenotype seen among PS patients. We performed whole-cell patch-clamp recordings on patient and control iPSC-derived neurons to address this question. Analysis of passive neuronal properties from the two PS subjects and two controls showed that PS patient-derived neurons have increased input resistance and a more depolarized resting membrane potential when compared to controls (Figures 5A,B). Additionally, the majority of both control and patient neurons fired spontaneously *in vitro* (Figure 5C), although there were no statistically significant differences in tonic firing (Figures 5D,E). We next examined different characteristics of evoked firing including the ability of neurons to fire repetitive APs, excitability, AP threshold and maximum firing rate (Figures 5F–H). PS patient-derived neurons showed a statistically significantly decreased threshold for initial AP generation (Figure 5J), consistent with their increased input resistance and depolarized resting membrane potential. No significant differences were seen between PS and control neurons for the other measures of evoked AP firing (Figures 5H,I,K).

**Figure 6:**
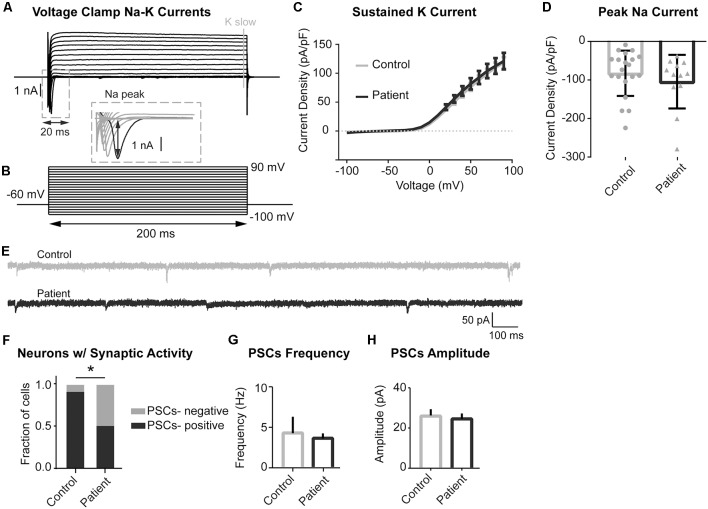
Sodium and Potassium conductances and synaptic activity in control and mutant neurons. (**A,B)** Example recording **(A)** and voltage-clamp protocol **(B)** used to evoke voltage-gated sodium and potassium conductances. **(A)** Series of the voltage clamp traces recorded from the control neuron. Inward fast currents, expanded in the inset window, demonstrate the presence of the sodium conductances. Peak Na current is labeled with black arrowheads. Outward slow conductances measured at the end of the voltage step (labeled with gray solid line) represent slow potassium currents. **(B)** Series of 200 ms voltage steps from −100 to +90 mV at 10 mV intervals, holding potential between steps is −60 mV. **(C)** Summary data of slow potassium conductances represented as Mean ± SEM current density (Y-axis) in function of voltage (X-axis); sample sizes: Control (solid gray line) *n* = 20, Patient 2 (solid black line) *n* = 12; multiple *t*-tests for each current step, adjusted *p* values n.s. **(D)** Summary data of peak sodium currents represented as Mean ± SEM current density; sample sizes: Control (gray bar) *n* = 20, Patient 2 (black bar) *n* = 12; Unpaired Student’s *t*-test, *F* = 0.7385, *p* = 0.3526 (n.s). **(E)** Example traces of recordings of spontaneous postsynaptic currents (PSCs) acquired in voltage-clamp mode with the membrane clamped at −60 mV. Control—gray, Patient 2–black. **(F)** Summary data of the proportion of neurons demonstrating spontaneous synaptic activity, data represented as a fraction of recorded cells with (black) and without (gray) detected PSCs; sample sizes: Control *n* = 19, Patient 2 *n* = 12; Fisher’s exact test, df = 2, *p = 0.0316. **(G)** Summary data of PSCs Frequency (only PSCs-positive cells), data represented as Mean ± SEM; sample sizes: Control (gray bar) *n* = 17, Patient 2 (black bar) *n* = 6; Mann–Whitney test, exact *p* = 0.5069 (n.s.). **(H)** Summary data of PSCs Amplitude (only PSCs-positive cells), data represented as Mean ± SEM; sample sizes: Control (gray bar) *n* = 17, Patient 2 (black bar) *n* = 6; Mann–Whitney test, *p* = 0.2863 (n.s.).

Voltage clamp experiments to study sodium and potassium conductances revealed no differences in currents between the PS patient and control neurons (Figures 6A–D). Interestingly, we did find a difference in spontaneous synaptic activity, with PS patient-derived cells displaying a much lower prevalence of spontaneous postsynaptic currents (sPSCs) than control cells, suggesting a delayed or altered network connectivity formation (Figures 6E,F). Neurons that did exhibit spontaneous synaptic activity did not differ between the two experimental groups in terms of sPSC frequency or amplitude (Figures 6G,H). Together, these findings indicate that PS neurons have an electrophysiological phenotype consistent with increased intrinsic excitability and decreased synaptic activity, both of which may relate to delayed neuronal maturation.

### *Strada* KO Mouse

Previous work has shown that germline *Strada* KO is associated with a perinatal lethal phenotype with death occurring by post-natal (P) day 2 (Veleva-Rotse et al., 2014). Our C57/Bl6N *Strada*−/− strain (del exon 9–13) showed reduced survival of *Strada*−/− pups within the first five post-natal days and thus out of more than 100 litters yielding both WT and heterozygous pups, only eight *Strada*−/− animals from eight distinct litters (five females, three males) survived past P5, four into adulthood; multiple *Strada*−/− mice were never found in the same litter. The oldest living *Strada*−/− mouse in our colony is 10 months old. All heterozygous (*Strada*+/−) mice were identical in appearance and behavior to wildtype animals with no obvious behavioral or motor phenotype and clinical seizures were not observed in either *Strada +/−* or *Strada −/−* mice. Of note, *Strada* +/− females had either litters that were smaller than WT (<3 pups) or no litters at all, despite constant breeding. Indeed, despite heterozygous/heterozygous and heterozygous/homozygous mating attempts (we did not generate enough viable *Strada*−/− adults for matings), *Strada*−/− pups were very infrequently found.

**Figure 7:**
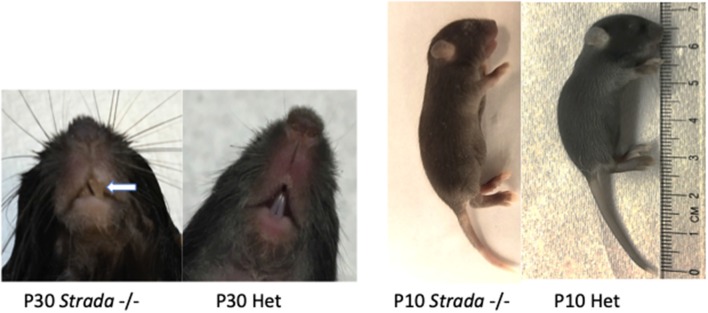
Effects of Strada loss on development. *Strada*−/− animals had a dysmorphic skull structure with domed appearance and maloccluded incisor teeth (arrow). The animal body size was consistently smaller than either WT or heterozygous mice (Het).

Overall observation of all *Strada*−/− mice revealed reduced body size compared to either *Strada*+/− or wildtype littermates. All *Strada*−/− animals had a dysmorphic skull structure with a domed appearance and maloccluded incisor teeth (Figure 7). The large incisors and malocclusion were seen in all eight *Strada*−/− mice and interfered with successful feeding. Veterinary medicine staff removed the incisors to facilitate feeding. *Strada*−/− mice were hypotonic with limited mobility in the cage, and when attempting to ambulate they exhibited marked tremor. Under direct observation, the pups had clear difficulties initiating and maintaining suckling in comparison with *Strada*+/− pups. All *Strada*−/− mice had properly formed anterior cruciate ligaments (ACL), unlike some PS patients who have congenital absence of the ACL (data not shown; Puffenberger et al., 2007).

**Figure 8:**
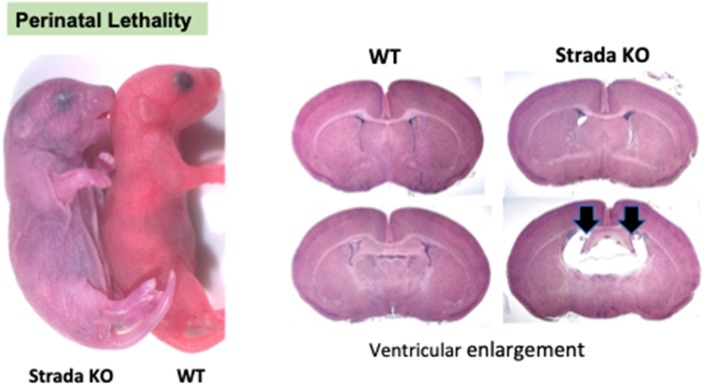
Perinatal lethality in *Strada* −/− mice. Left, a comparison of *Strada*−/− to wildtype (WT) mouse pup. Right, evidence of ventricular enlargement (arrows) in P10 *Strada*−/− mice.

**Figure 9:**
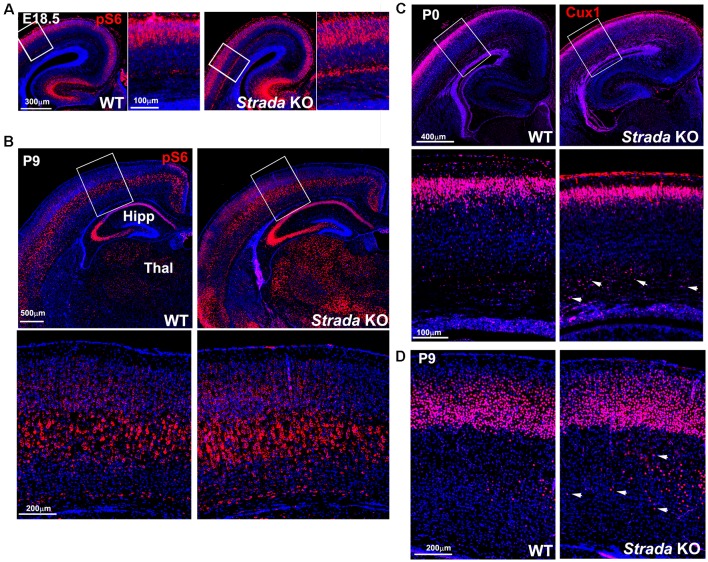
Enhanced ribosomal S6 phosphorylation (P-S6) in *Strada*−/− mice evident at E18.5 **(A)** and P9 **(B)** compared with WT mouse. There is enhanced P-S6 immunoreactivity in the cerebral cortex deep layers, the thalamus (Thal), and the hippocampus (HIPP). Box depicts inset below, which shows P-S6 labeled neurons. **(C)** At P0, more Cux1 labeled late-born neurons (Layer II–IV) are accumulated in the intermediate zone of KO (arrows) compared with WT. **(D)** At P9, more Cux1 positive neurons are found in the deeper layer of the white matter of *Strada* KO (arrows) compared with WT.

Neuropathological analysis of *Strada*−/− mice (*n* = 5; ages P2–P20) demonstrated that the brains were enlarged with ventriculomegaly, but there was a normal gross structure of the hemispheres (Figure 8). Given our small *Strada*−/− sample size, differences in brain weights between *Strada*−/−, heterozygous, and WT animals could not be determined. There were enhanced numbers of cells immunoreactive for P-S6 (Ser 240/244) found in late embryogenesis (embryonic day 18.5; Figure 9A) that were also seen throughout all cortical layers, the thalamus, and hippocampus in the *Strada*−/− mice compared with *Strada*+/− and WT mice at P9 (Figure 9B). At P0, more Cux1 positive late-born neurons are found in the deeper layer or intermediate zone, suggesting that there may be abnormal migration of late-born neurons (Figure 9C). Consistently, displaced ectopic Cux1 positive neurons within the deeper layer or subcortical white matter were observed in *Strada*−/− brains analyzed at P9 as compared to littermate control WT (Figure 9D). These mirrored observations in human PS brain tissue (Puffenberger et al., 2007).

## Discussion

Using several model systems, we demonstrate that loss of *STRADA* in human iPSC-derived neurons and mature mouse neurons *in vivo* causes activation of mTOR pathway signaling, placing PS squarely as a mTORopathy. *STRADA* loss causes enhanced cell size in all systems studied. We show that germline KO of *Strada* effectively models many of the histological features of human PS with enlarged brain size, ventriculomegaly, and heterotopic neurons in the subcortical white matter. Finally, iPSC-derived neurons from PS patients exhibit subtle electrophysiological abnormalities including increased input resistance, a more depolarized resting membrane potential, and a decreased threshold for initial AP generation.

We show that the PS patient-derived iPSCs and neurons do not express STRADA and exhibit enhanced mTOR activation. These findings are consistent with our previous *in vitro* and *in vivo* mouse models, demonstrating that knockdown of STRADA leads to enhanced mTOR signaling (Orlova et al., 2010; Parker et al., 2013). Thus, these cells provide an attractive *in vitro* system to study mTORopathies. Neurons derived from PS patient iPSCs recapitulate many of the abnormal features observed in fibroblasts, lymphocytes, and fixed brain tissue from PS patients (Orlova et al., 2010; Parker et al., 2013). These iPSC and neuronal lines join a small list of lines generated from patients with a mTORopathy, including TSC and individuals with focal cortical dysplasia linked to *DEPDC5* mutations (Blair et al., 2018; Winden et al., 2019). Providing *in vitro* models of human mTORpathies is a major challenge because of the mutational mechanisms causing these disorders. Thus, an important feature of our cells is that we can generate neurons with a causative gene deletion from fibroblasts. Our PS iPSCs and neurons are unique as other identified mTORopathies result from heterozygous mutations that require the subsequent loss of heterozygosity *via* a “second hit” somatic mutations; the PS subject-derived neurons with homozygous *STRADA* loss-of-function deletions provide a unique window into mTOR activation in a homogeneous genomic background. In our studies, iPSC-derived neurons lacking STRADA show cytomegaly, likely related to mTOR hyperactivation (Orlova et al., 2010), but do not have defects in differentiation. A recent study examining hESCs with heterozygous or homozygous *TSC2* loss-of-function generated by zinc-finger nuclease-mediated gene disruption showed that homozygous loss of TSC2 produced cytomegaly as well as increased dendritic complexity (Costa et al., 2016). Thus, while a common manifestation of mTOR hyperactivation is increased cell size, other structural, phenotypic alterations may be unique to each genotype. The current results show that PS derived neurons reflecting germline STRADA deletion provide a reproducible human cell system to study the effects of loss of STRADA on mTOR signaling.

*Strada* +/− mice had no phenotype. These observations parallel human heterozygous PS individuals (patient parents) who are neurologically normal (Puffenberger et al., 2007; personal clinical observations, P. Crino). A previously reported germline *Strada* knockout mouse strain exhibited subtle alterations in cortical lamination and changes in axonal outgrowth and exhibited perinatal mortality (Veleva-Rotse et al., 2014). Our *Strada*−/− mice also exhibited early lethality (from yet undefined causes) but a few animals survived to adulthood. These mice show limited mobility, tremor, and enhanced brain size. Spontaneous seizures were not observed but our sample size was too small to assess seizure phenotype. As in human PS brain histopathology, overt malformations of cortical development (MCD) was not observed in the *Strada*−/− mice, however, CUX-1 labeled heterotopic neurons were seen in the subcortical white matter which has been reported in human PS suggesting a migratory defect in the cerebral cortex. STRADA function has been implicated in the establishment of neuronal polarity and cell migration and is necessary for intact Golgi apparatus formation (Parker et al., 2013; Rao et al., 2018).

Our results suggest that STRADA loss slightly increases cell-autonomous excitability, which may contribute to an epileptic phenotype. In contrast, synaptic activity is reduced in PS neurons, consistent with a previous report of *TSC2* deletion in hESCs-derived neurons, which demonstrated decreased synaptic excitability (Costa et al., 2016). Both enhanced intrinsic excitability and a paucity of synaptic activity may reflect delayed maturation of PS-derived neurons. Ongoing research will define whether these findings relate to the severe epilepsy phenotype in PS patients. Another pertinent issue is that interneurons may be required for manifestation of the network hyperexcitability phenotype, and such a phenotype was not reflected in our *in vitro* model which consisted primarily of excitatory cortical-like neurons. Future studies should address these potential limitations by focusing on mixed excitatory and inhibitory populations in 2-D or brain organoid culture systems, the latter of which can be maintained for prolonged periods to promote maturation and also provides a 3-dimensional network architecture. These findings thus provide new and compelling evidence that STRADA loss by itself can confer a hyperexcitability phenotype, although the underlying abnormalities leading to increased spontaneous activity require further investigation. Future studies should focus on the downstream consequences of STRADA loss to uncover epilepsy mechanisms in PS that would likely apply to other mTORopathies and lead to the development of novel therapies.

## Data Availability Statement

The raw data supporting the conclusions of this article will be made available by the authors, without undue reservation, to any qualified researcher.

## Ethics Statement

The studies involving human participants were reviewed and approved by University of Pennsylvania Human Subjects Committee. Written informed consent to participate in this study was provided by the participants’ legal guardian/next of kin. The animal study was reviewed and approved by University of Pennsylvania Animal Care Committee. The animal study was reviewed and approved by University of Pennsylvania, Temple University, and University of Maryland School of Medicine Institutional Animal Care and Use Committees.

## Author Contributions

LD, YL, GM, AS, and SV: iPSC derived neurons, westerns, data analysis, and manuscript writing. AB, MB, and PI: generated CRISPR lines/validation, immunocytochemistry, and mouse KO strain work. SK and UK: mouse KO strain work. WP: identified PS patients, procured, maintained and analyzed fibroblasts for iPSCs. KG, FH, GP, and MS: electrophysiology and data analysis. PC and JP: conception of the study and experimental design, data analysis, and manuscript writing. LD, KG, PI, AB, MB, YL, GP, SV, AS, WP, SK, UK, FH, GM, MS, JP, and PC contributed to the manuscript revision, read, and approved the submitted version.

## Conflict of Interest

The authors declare that the research was conducted in the absence of any commercial or financial relationships that could be construed as a potential conflict of interest.

## Supplementary Material

FIGURE S1 Transgenic germline construct for generation of *Strada*^−/−^ mice.

FIGURE S2 Southern blot confirms that transformed ES cells carry the Strada transgenic construct.
