# Supplementary figures and images for: Multimodal Analysis of STRADA Function in Brain Development
Source: Front Cell Neurosci. 2020 May 8;14:122. doi: 10.3389/fncel.2020.00122 (PMC7227375; doi:10.3389/fncel.2020.00122)

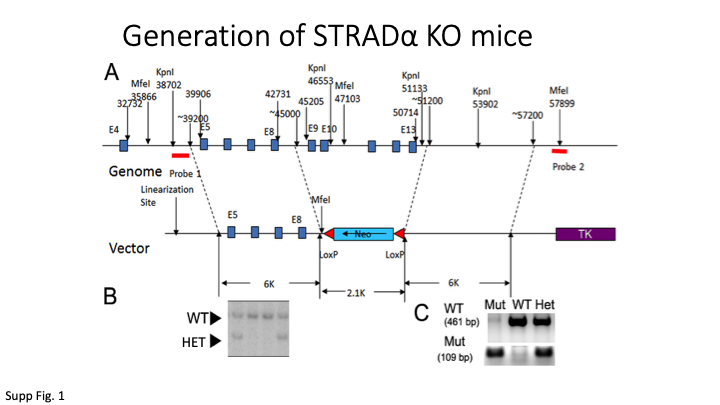

Supplement: FIGURE S1 — Transgenic germline construct for generation of Strada−/− mice. [file Image_1.TIFF]

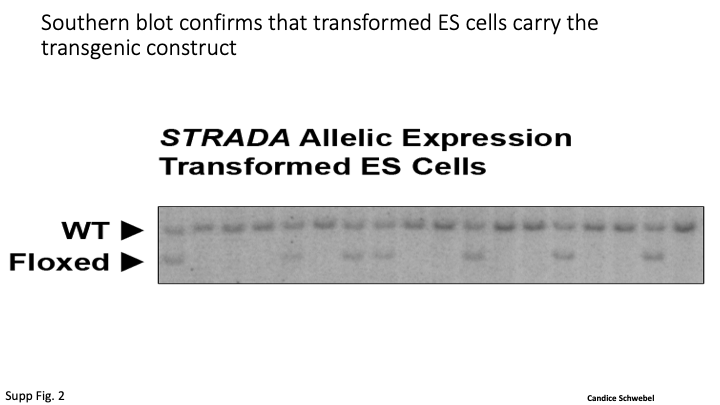

Supplement: FIGURE S2 — Southern blot confirms that transformed ES cells carry the Strada transgenic construct. [file Image_2.TIFF]
